# The Effect of Organic Loading and Mode of Operation in a Sequencing Batch Reactor Producing PHAs from a Medium Corresponding to Condensate from Food Waste Drying

**DOI:** 10.3390/polym17243270

**Published:** 2025-12-09

**Authors:** Konstantina Filippou, Konstantina Diamantopoulou, Melisa Gatzia, Ioanna Ntaikou, Konstantina Papadopoulou, Gerasimos Lyberatos

**Affiliations:** 1 School of Chemical Engineering, National Technical University of Athens, Iroon Polytechneiou 9, Zografou, 15780 Athens, Greece; filippoukonstandina@gmail.com (K.F.); ch19001@chemeng.ntua.gr (K.D.); ch20052@mail.ntua.gr (M.G.); kpapado@chemeng.ntua.gr (K.P.); 2 Institute of Chemical Engineering Sciences (ICE-HT), Stadiou Str., Platani, 26504 Patras, Greece; intaikou@upatras.gr; 3 Department of Civil Engineering, University of Patras, University Campus, 26500 Patras, Greece

**Keywords:** polyhydroxyalkanoates, sequencing batch reactor, organic loading, settling

## Abstract

This study evaluated polyhydroxyalkanoates (PHAs) production from a medium corresponding to the condensate derived from food waste drying, using a mixed microbial culture in a 15 L Sequencing Batch Reactor (SBR). The reactor operation comprised two distinct periods to investigate the impact of varying organic loading rates on biomass performance and polymer accumulation. In Period 1, when the soluble Chemical Oxygen Demand (sCOD) was 6.8 ± 1.4 g/L, efficient nitrogen limitation promoted complete urea consumption and stable biomass growth, yielding higher intracellular PHA accumulation (11.74 ± 6.01%). The microbial community exhibited a balanced copolymer production (HB:HV ratio of approximately 54:46). Conversely, Period 2, characterized by higher organic loads (sCOD 12.1 ± 2.9 g/L), displayed incomplete urea utilization, reduced biomass viability, and significantly lower PHA accumulation (5.26 ± 2.53%). A second set of experiments aiming at the assessment of the impact of operation mode (with and without inclusion of a settling phase) demonstrated that removal of settling leads to a stable long-term steady-state operation with enriched PHA-accumulating bacteria and increased polymer storage capacity.

## 1. Introduction

The increasing dependence on fossil-based carbon resources has raised serious environmental concerns due to their unsustainable extraction, their widespread utilization, and their significant contribution to greenhouse gas emissions, all of which intensify global climate change. Additionally, the petrochemical industry relies heavily on fossil carbon for manufacturing plastics that are highly persistent and non-biodegradable, causing further environmental pollution [1]. Petroleum-derived plastics, characterized by complex chemical structures, can persist in natural environments for centuries, resisting both mechanical and chemical breakdown, and contributing to the alarming rise in microplastics in the ecosystems [2]. Improper disposal results in severe ecological consequences, including soil contamination, groundwater pollution through leachate infiltration during landfilling, the release of harmful volatile compounds during combustion, and disruptions to natural ecosystems [1]. Furthermore, the degradation of larger plastic items leads to the formation of microplastics that are easily dispersed in soils, waterways, and living organisms, further exacerbating ecological disruption [3]. Thus, to address these pressing issues, the development of sustainable alternatives to mitigate the long-term environmental impact of fossil-derived materials is required.

Biobased and biodegradable plastics have emerged as a promising alternative to conventional plastics, by maintaining desirable material properties of traditional plastics, while alleviating their environmental drawbacks [4]. Among these, polyhydroxyalkanoates (PHAs) are of particular interest because they are microbially synthesized and recovered via solvent extraction and/or cell lysis [5]. PHAs accumulate intracellularly in the form of granules under nutrient-limited, carbon-rich conditions [6]. They exhibit physicochemical properties comparable to polypropylene, while remaining biodegradable in both aerobic and anaerobic conditions [7]. Their properties vary depending on their monomeric composition, rendering them suitable alternative raw materials for the packaging and food industries, as well as materials for agricultural applications.

Despite the growing interest in the commercialization of bio-based biodegradable polymers over the past decades, the current market share of PHAs is very small. Only 25,200 tons were produced in 2019, accounting for 1.2% of the overall bioplastic market [5]. This is because several challenges must still be addressed for PHAs to achieve competitive market viability. Currently, industrial PHA production relies mostly on pure cultures, capable of accumulating PHAs intracellularly, accounting for up to 90% of their cell dry weight. 25.3 kt of PHA are produced annually at a market value of $7.0/kg. However, the need for high-purity substrates, sterile conditions, and refined biotechnological processes significantly increases the costs of production [8]. As a result, the production cost of PHAs remains significantly higher (varying between $4000 and $15,000 per metric ton (MT), in comparison with $1000 to $1500 per MT of fossil-derived plastics [9].

Industrial-scale production of PHAs requires evaluating techno-economic aspects, raw material supply, and target markets for the products, making pilot-scale facilities essential for bridging laboratory research and commercial implementation [10]. Scaling up biopolymer production remains challenging due to the high costs associated with both upstream and downstream processes [9]. Microbial growth efficiency, nutrient supply, and recovery methods all influence large-scale feasibility, prompting ongoing efforts to optimize fermentation processes, bioreactor designs, and strain selection [11]. Reducing feedstock costs is particularly important; mixed microbial cultures (MMC) provide major advantages by operating in non-sterile environments, while utilizing diverse complex organic substrates [12]. Using waste materials, such as agricultural residues, industrial by-products, and food waste, as feedstocks further enhances economic viability, since their fermentation generates volatile fatty acids (VFAs) that are suitable as direct PHA precursors [13,14].

Sequencing batch reactors (SBRs) are commonly used for PHA production because they offer low contamination risks, high operational flexibility, and tight control of the conditions to maintain microbial stability [15]. They can combine all the processing steps, e.g., feeding, aerobic reaction, and withdrawal phases, creating feast/famine conditions that favor PHA accumulation, in a single tank. Thus, the use of an SBR, operating “in time” rather than “in space”, is an ideal solution where space is limited [16].

Despite their broad use, the effect of SBR operational modes, especially the settling phase, remains insufficiently documented. Most studies include a biomass settling phase, which causes biomass to progressively accumulate until removal is necessary for PHA recovery [17,18,19]. As biomass builds up, part of the accumulated PHAs may be consumed, reducing process efficiency [20]. In order to avoid the need for interrupting process operation for PHA harvesting, it is necessary to include a separate biomass removal phase in each SBR cycle if a steady-state, uninterrupted operation is to be established. As soon as steady-state conditions are reached, the active biomass concentration, PHA content, and substrates are kept constant [21]. This approach is mainly useful for studying microbial physiology and optimizing nutrient composition under stable conditions [22]. In order to avoid the two extra phases (settling and biomass removal), PHA harvesting may take place from the SBR effluent. This simplifies the SBR operation, while also ensuring that nondegradable solids, present in the feed, do not accumulate in the reactor.

The aim of this work is to investigate PHA production using an MMC operated under dynamic aerobic feeding in a 15 L SBR. PHA accumulation in the system is driven by nitrogen limitation. A synthetic medium mimicking the liquid fraction produced during food-waste drying [23,24] is used as the carbon source, while urea provides nitrogen. Because condensate is naturally low in nitrogen, it serves as an excellent substrate for establishing alternating nitrogen- and carbon-limited conditions that favor PHA synthesis. The study evaluates the impact of different organic loading rates and two operational modes, e.g., one incorporating a biomass settling phase and one omitting it. Removing biomass without settling represents a significant alternative, as it may promote the selection of highly active PHA-accumulating organisms, enable faster achievement of steady-state conditions, reduce PHA consumption by the culture, and ultimately enhance overall process effectiveness.

## 2. Materials and Methods

### 2.1. Experimental Setup and Operational Parameters

**Figure 1:**
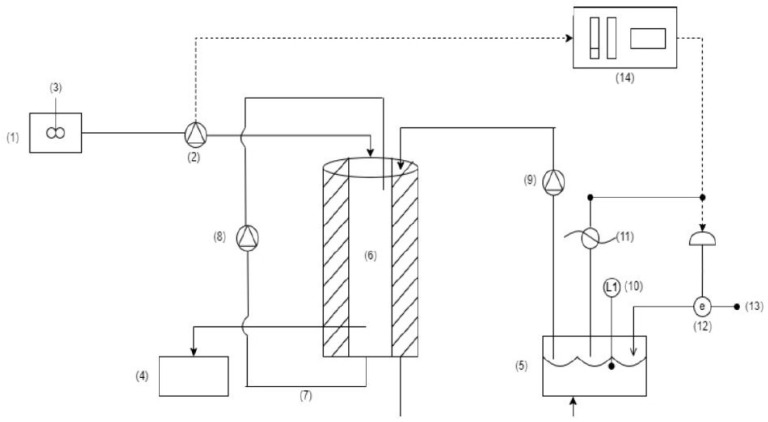
Schematic Diagram of the Experimental Setup (1) Influent Tank (2) Lift Pump (3) Stirrer with Thermocouple (the thermocouple is not depicted in the schematic) (4) Water Bath Reservoir (5) Reaction Zone and Tank (6) Mixed Liquor Recirculation (7) Mixed Liquor Recirculation Pump (8) Submersible Water Recirculation Pump (9) Level Indicator (10) level Indicator (11) Electric Heater (12) Electric Valve for Water Losses (13) Water Supply from the Main Network (14) Control Panel.

A double-coated SBR with a total volume of 15 L and a working volume of 12 L was constructed from Plexiglas and operated under non-sterile conditions, as shown in Figure 1. The reactor was kept at room temperature (27 °C) via warm water recirculation in its outer jacket. Agitation was performed via aeration, which was supplied by a fine bubble diffuser, ensuring dissolved oxygen (DO) above 2 mg/L, during the reaction phase. A synthetic medium containing either a carbon or nitrogen source, a basal synthetic medium, and a trace element solution was used in each feeding cycle. For the carbon-rich feeding (PHA accumulation phase), a synthetic solution with a composition corresponding to the liquid fraction produced from drying/shredding of food waste (condensate) [23,24] was composed of acetic acid (27% sCOD), butyric acid (27% sCOD), propionic acid (6% sCOD), ethanol (20% sCOD), glucose (15% sCOD), and lactic acid (5% sCOD). The synthetic medium was used instead of real condensate to secure stable, reproducible operation [24]. Urea served as the nitrogen source in the nitrogen-rich phase (growth phase). Both solutions were supplemented with MgSO_4_·7H_2_O, CaCl_2_·2H_2_O, K_2_HPO_4_, KH_2_PO_4_, and trace elements.

Two independent sets of experiments were conducted. The first experiment aimed at evaluating the impact of organic load on the SBR performance, including a settling phase to ensure biomass retention and to isolate the influence of carbon load. For a start-up, a pre-acclimated culture in a draw-and-fill reactor at lab-scale, using a synthetic medium similar to the reactor feed, was used [19]. The start-up culture volume for this first set of experiments was 30% *v*/*v.* To ensure that microorganisms maintained their capability of storing carbon as PHAs, dynamic feeding conditions were applied. The experiment can be divided into three periods as shown in Table 1. The Batch Period was conducted to acclimate the microbial culture to the operating conditions of the new reactor. During Period 1, the dynamic feeding conditions were applied under nutrient stress. The mean soluble COD (sCOD) at the start of each C cycle, when adding 2/3 feed in the reactor for this period, was 6.8 ± 1.4 g/L. Period 2 was conducted in the same reactor as a continuation of the previous periods, to study the behavior of the system by increasing the organic loading of the feed. The carbon-to-nitrogen (C:N) ratio was set at 100:1. The overall operation lasted 177 days.

**Figure 2:**
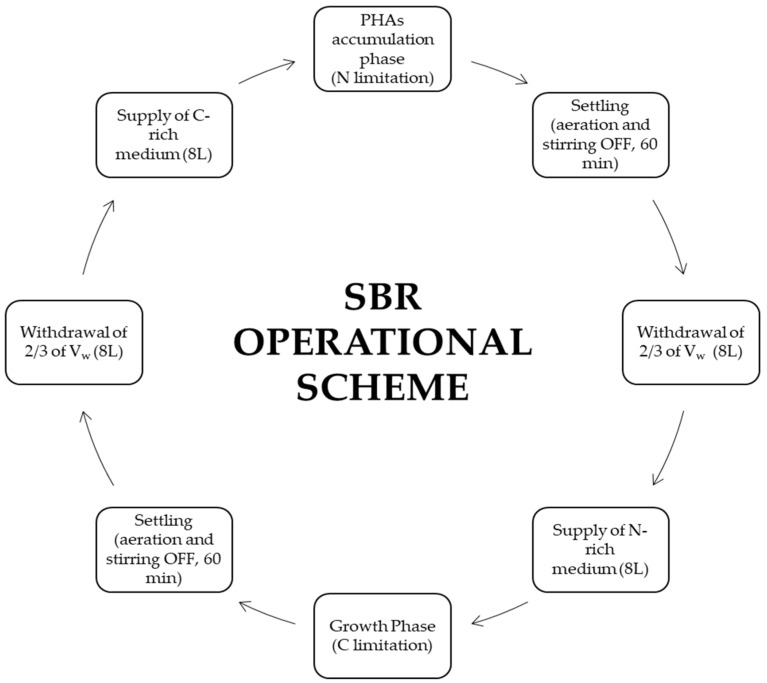
Schematic representation of the operational scheme of the SBR. The system alternated between aerobic growth (N cycle) and aerobic accumulation (C cycle), each followed by a settling phase and withdrawal of two-thirds of the supernatant with media supply.

The SBR operated as shown in Figure 2, alternating a growth phase (N cycle), in which the urea medium was supplied, and a PHA accumulation phase (C cycle), in which the condensate was supplied. The settling phases lasted 60 min, with an 80 min interval for supernatant withdrawal (2/3 of the SBR liquid content, 8 L) and replacement with an equal volume (8 L) of the next medium. The reaction phases (growth and accumulation cycles, N and C, respectively) followed a biweekly schedule (2d N–2d C–3d N–2d C–2d N–3d C).

In order to investigate the impact on biomass retention and PHA accumulation without the settling phase, a second experiment was carried out. The second set of experiments aimed at comparing the SBR operation with and without the inclusion of a settling phase. During this configuration, the operational cycle of the SBR consisted also of consecutive growth and accumulation phases, while omitting; however, the 60 min settling phase. The omission of the settling phase altered biomass washout dynamics, since no intentional biomass withdrawal was carried out. Biomass loss occurred solely through natural washout via the 2/3 withdrawal under continuous aeration. Therefore, the concentration of solids was influenced by the balance between microbial growth, PHA accumulation, and the hydraulic washout. This provided a basis to compare operational robustness and polymer yields under both modes of operation.

For this experiment, an enriched aerobic mixed culture was developed using as an inoculum sludge from the recirculation of the secondary clarifier of the Wastewater Treatment Plant of Lykovrisi, Attiki, Greece, during start-up. The inoculum concentration in the reactor upon start-up was set at 30% *v*/*v*, as in the first experiment. The experimental organic load for this experiment was 6.5 ± 0.7 g/L. The carbon-to-nitrogen (C:N) ratio was 100:1, and the overall operation lasted 110 days. The study can be divided into three periods. The three experimental periods of the SBR operation to this end are shown in Table 2. The Batch Period was conducted to acclimate the microbial culture to both the carbon and nitrogen sources. Period 1 was carried out with settling in order to build up sufficient biomass concentration, before Period 2, which involved no settling. Period 2 was conducted in the same reactor as a continuation of the previous periods, to study the behavior of the system by removing the settling phase.

### 2.2. Analytical Techniques

The analysis of sCOD, Total Suspended Solids (TSS), and Volatile Suspended Solids (VSS) was conducted according to Standard Methods for the Examination of Water and Wastewater [25]. The pH was measured using a digital pH-meter (WTW INOLAB PH720, Weilheim, Germany). For the quantification of Volatile Fatty Acids (VFAs), 1 mL of sample was acidified with 30 μL of 20% H_2_SO_4_ and analyzed via a gas chromatograph (SHIMADZU GC-2010 plus, Kyoto, Japan). The chromatograph was equipped with a flame ionization detector and a capillary column (Agilent technologies, 30 m × 0.53 mm ID × 1 μm film, HP-FFAP) using an autosampler (SHIMADZU AOC-20 s, Kyoto, Japan). Ethanol, glucose, and lactic acid were measured via High Performance Liquid Chromatography (HPLC) with an Agilent Technologies (Santa Clara, CA, USA) 1260 Infinity II HPLC, using the column Agilent Hi-plex H of 300 mm  ×  7.7 mm (Santa Clara, CA, USA), as described in Kiskira et al. 2023 [26]. Total Nitrogen (TN) was measured with a Shimadzu TN-L analyzer (Shimadzu, Kyoto, Japan; TOC-VCHS and SSM-5000 module). The PHAs detection method was based on the simultaneous extraction and transesterification of PHAs. The frozen and lyophilized biomass pellets in glass tubes were used for determining the concentration and monomeric composition of the PHAs by gas chromatography (SHIMADZU GC-2010, Kyoto, Japan) with flame ionization detection and a capillary column (MEGA 5HT, 30 m × 0.25 mm ID × 0.25 μm film, Legnano, Italy). The methodology used was acidic methanolysis as described in Oehmen et al. (2005) [18,27]. Pure poly (3-R-hydroxybutyrate-co-3-R-hydroxyvalerate) (PHBV) copolymer with a 3-R-hydroxyvalerate (3HV) content of 8 mol% from Sigma-Aldrich (St. Louis, MO, USA) was used for calibration; thus, the contents of 3-R-hydroxybutyrate (3HB) and 3HV were determined; PHAs were defined as the sum of 3HB and 3HV.

## 3. Results and Discussion

### 3.1. First Set of Experiments for Assessing the Effect of Organic Load

The average concentration of sCOD at the start of the carbon cycles was 6.8 ± 1.4 g sCOD/L during Period 1, while in Period 2, the initial sCOD concentration was 12.1 ± 2.9 g sCOD/L.

**Figure 3:**
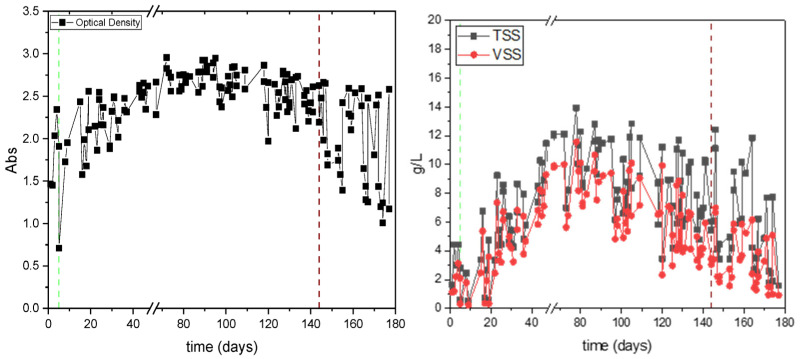
Optical density (**left**), TSS, and VSS (**right**) in the PHAs-producing SBR in the experiment assessing the effect of organic load. The green dashed line represents the transition from initial batch operation, where both carbon and nitrogen were supplied to Period 1, when nutrient stress conditions (nitrogen limitation) were applied. The red dashed line marks the transition from Period 1 to Period 2, characterized by an increased organic loading rate.

As shown in Figure 3, optical density (OD), TSS, and VSS follow similar trends throughout the reactor operation. During Period 1, OD ranged from 1.7 to 2.96, while in Period 2, from 1.01 to 2.66. In both periods, OD, TSS, and VSS decrease at the start of each cycle due to sedimentation and removal of the supernatant, followed by increases during the carbon (accumulation) and nitrogen (growth) phases. Higher values were generally observed at the carbon phases, when microorganisms consume the carbon source to synthesize and store PHAs. During the first 17 days, the OD showed considerable variation before stabilizing. After the 17th day, a clear increasing trend is observed. The biomass reached a maximum level, after which part of it was removed to maintain stable TSS and VSS values. In Period 2, the OD stabilized at lower values and exhibited a higher variation during the cycle. This reduction is due to the inability of certain microorganisms to survive under conditions of nutrient deprivation. According to the literature, poor separation through sedimentation is associated with cell lysis and a reduction in PHA granules [28]. Microorganisms that cannot accumulate carbon in the form of PHAs are ultimately removed along with the supernatant after sedimentation [29].

TSS and VSS measurements confirm these trends. In more detail, during the carbon phase, a significantly greater increase in solids is noted, indicating that carbon is utilized for energy conservation and accumulation of intracellular products, such as PHAs [30]. At the end of the nitrogen phase, a smaller increase in solids is observed, which is attributed to the growth of microorganisms that have survived and are capable of biosynthesizing PHAs, consuming the carbon they have accumulated along with nitrogen. During Period 1, a trend of increasing suspended solids is observed during the first 50 days of operation, and from that point on, biomass was periodically removed after settling so as to maintain a more or less constant solids concentration in the reactor. In Period 2, larger fluctuations in suspended solids concentrations were observed, with a notably smaller increase in biomass during the nitrogen cycle, indicating that there was insufficient intracellular carbon in the biomass to be utilized during the growth phase. The average concentrations of suspended solids were 7.72 ± 3.21 g TSS/L and 6.01 ± 2.91 g VSS/L in period 1, and 5.65 ± 3.06 g TSS/L and 3.26 ± 1.84 g VSS/L in period 2, respectively.

Finally, the ratio of VSS/TSS was 0.75 ± 0.09 in period 1 and 0.57 ± 0.05 in period 2. The drop in this ratio during the second period is due to an increase in inorganic substances [31]. In both cases, the VSS/TSS ratio suggests that the developed microbial biomass was healthy, as it aligns with the stable values of this ratio, indicating that progressive mineralization did not occur despite the long sludge retention time, and that the particulate content mainly consisted of bacterial products [31,32].

**Figure 4:**
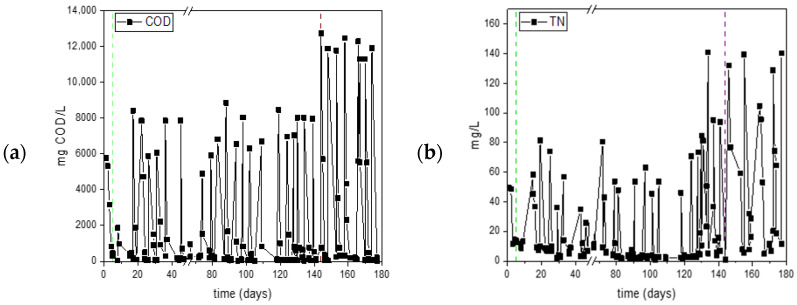
Consumption of sCOD (**a**) and TN (**b**) in the PHAs producing reactor SBR in the experiment assessing the effect of organic load. The green dashed line represents the transition from initial operation, where both carbon and nitrogen were supplied to Phase 1, at which point nutrient stress conditions (nitrogen limitation) were applied. The red dashed line marks the transition from Period 1 to Period 2, characterized by an increased organic loading rate.

The measurements of sCOD and TN during the operational period of the SBR are presented in Figure 4. At the end of each carbon cycle, significant sCOD reduction (90% in Period 1 and 87% in Period 2) was observed, promoting limitation conditions for subsequent cycles. During the nitrogen cycle, sCOD levels remained low, with occasional increases, likely due to the release of soluble organic matter from cell lysis [33]. The mean concentration of nitrogen at the beginning of each cycle was 66 ± 24 mg N/L in Period 1 and 124 ± 39 mg N/L in Period 2. During Period 1, urea was completely consumed in the nitrogen cycle, confirming the presence of sufficient intracellular carbon in the biomass. On the contrary, in Period 2, urea was not completely consumed during all nitrogen cycles, resulting in limited biomass growth and nitrogen availability in the carbon cycle. The inability to consume urea by the biomass indicates the lack of sufficient accumulated PHAs and reduced survival of microorganisms. This observation is consistent with findings in the literature, where limited availability of intracellular carbon reserves has been shown to impair the microbial community’s resilience and reduce nutrient acclimatization efficiency [34,35]. Furthermore, the detection of residual nitrogen at the start of carbon phases emphasizes insufficient nitrogen limitation, a critical factor influencing effective PHA synthesis. As reported previously, nitrogen limitation is crucial for inducing the metabolic shift necessary for substantial PHA accumulation; conversely, partial or suboptimal nitrogen stress results in lower polymer production and compromised microbial productivity [36]. Consequently, the conditions established during Period 2 appear inadequate to maintain optimal nutrient stress conditions, hindering the biomass’s capacity for efficient PHA synthesis and accumulation [37].

**Figure 5:**
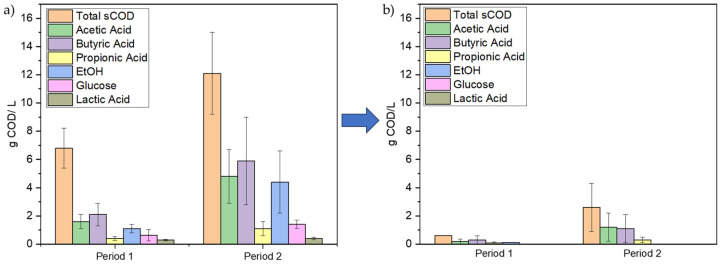
Mean concentration of total sCOD and each carbon source comprising the organic load of the synthetic carbon feed during the beginning of the carbon cycle (**a**) and the end of it (**b**) during both experimental Periods for PHAs production in the experiment assessing the effect of organic load.

The mean concentration of each carbon source in the synthetic media during the carbon cycle, e.g., VFAs-acetic, propionic, and butyric acid, as well as ethanol, glucose, and lactic acid, is shown in Figure 5. At the onset of the carbon cycle (Figure 5a), the mean concentrations of acetic, propionic, and butyric acid in Period 1 were 1.6 ± 0.5 g COD/L, 0.4 ± 0.1 g COD/L, and 2.1 ± 0.9 g COD/L, respectively, whereas in Period 2, these values increased to 4.8 ± 1.9 g COD/L, 1.1 ± 0.5 g COD/L, and 5.9 ± 3.2 g COD/L. Similarly, in period 1, ethanol, glucose, and lactic acid were 1.1 ± 0.3 g COD/L, 0.5 ± 0.4 g COD/L, and 0.3 ± 0.1 g COD/L, respectively. These values increased during Period 2 (4.4 ± 2.1 g COD/L, 1.4 ± 0.3 g COD/L, and 0.4 ± 0.1 g COD/L, respectively. Although complete depletion of VFAs is not achieved in every carbon cycle, the overall consumption patterns remain consistent between the two periods. Table 3 summarizes the mean COD reduction for each carbon source, indicating generally higher substrate uptake during Period 1 for VFAs, while ethanol, glucose, and lactic acid are completely consumed in Period 2. The variations in VFAs and substrate consumption can be attributed to the distinct metabolic pathways of each compound and the biochemical properties of the enzymes involved. Moreover, this may also be attributed to the presence of residual nitrogen, which, as shown in the study by Albuquerque et al. [38], supports both PHA accumulation and limited microbial growth, potentially enabling more efficient carbon source utilization by both PHA-storing and non-PHA-storing populations.

In general, VFAs’ consumption is influenced by the carbon source characteristics and the mixed culture microbial composition. Notably, according to the literature, ethanol, glucose, and lactic acid can serve as precursors for the formation of HB and HV monomers in the presence of propionic acid [39,40,41].

**Figure 6:**
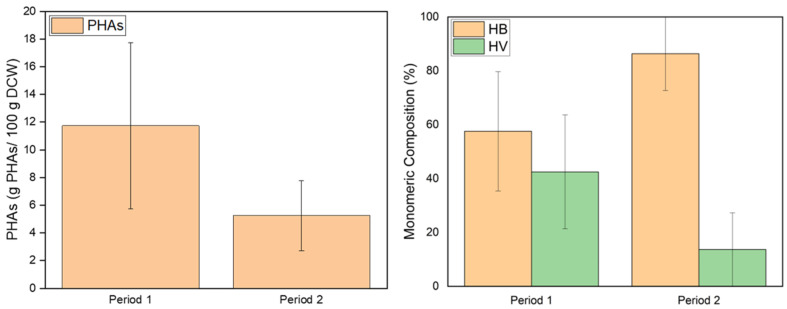
Average yield of PHAs during each period of operation of SBR (**left**) and monomeric composition of the PHAs (**right**) in the experiment assessing the effect of organic load.

Figure 6 shows the average accumulation of PHAs during the two operational periods of SBR. Specifically, 11.74 ± 6.01% PHAs is achieved in Period 1, and an accumulation of 5.26 ± 2.53% PHAs in Period 2. The yields obtained in the current work fall on the lower side of the range typically reported for MMC systems. Specifically, previous studies, using different enrichment and accumulation techniques for MMCs, utilizing industrial waste streams as carbon sources, have reported PHA yields between 7.6 and 76 wt% pilot-scale PHA production [42]. The observed reduction in PHA accumulation under higher organic loading can be mechanistically explained by the relationship between nitrogen availability and carbon source utilization. In Period 1, complete urea consumption ensured nitrogen limitation, e.g., nutrient stress conditions, activating the metabolic switch toward PHAs accumulation, which was not the case in Period 2. On the contrary, residual nitrogen during that time disrupted this stress response, leading to incomplete activation of PHAs synthase pathways [43]. This suggests that microbial competition under high organic load favored non-PHA-storing populations, therefore weakening both polymer yield and diversity. It is demonstrated that suboptimal nutrient stress leads to reduced storage capacity in MMCs even when VFAs remain available.

Regarding the monomeric composition of the PHAs, the production of either the copolymer polyhydroxy-butyrate-valerate (PHBV) or the homopolymers polyhydroxy-butyrate (PHB) and polyhydroxy-valerate (PHV) is achieved. In Period 1, the HB:HV ratio was (53.54 ± 22.13):(46.46 ± 21.14), while in Period 2 it was (86.34 ± 19.44):(13.66 ± 19.44). The high HV content in Period 1 is attributed to propionic acid being consumed to a greater extent than the other volatile fatty acids, especially butyric acid. HV synthesis is highly dependent on odd-chain VFAs such as propionate and valerate, which serve as direct precursors for HV formation. In contrast, in Period 2, which exhibited the lowest consumption rates, the HV content decreases [39]. Particularly, where the organic load of the feed increased while maintaining its composition, the microbial community favored HB synthesis. This may be attributed to the reduced uptake of propionate, a direct precursor for HV monomers, and the possible rapid consumption of propionate by non-PHA-storing microbial populations for energy generation and biomass synthesis, reducing its availability for PHA accumulation [44]. This phenomenon, coupled with competition among microbial consortia, leads to a metabolic shift favoring HB-rich polymers rather than HV-dominant copolymers.

### 3.2. Second Set of Experiments for Assessing the Impact of Including or Not Including a Settling Phase

The initial seed for this operation mode was an aerobic sludge from the WWTP of Lykovrisi, Attiki. Upon the startup of the reactor, both nitrogen and carbon sources were provided. The sequential limitation, i.e., starting with carbon limitation and solely nitrogen supply, was subjected to the system on the 4th day of operation. The operation of the reactor was divided into two distinct periods in order to assess the impact of the settling phase on system performance. During Period 1, the reactor was operated with a settling phase that lasted 60 min, while in Period 2, this step was omitted. During the second period, withdrawal and supply of new media were performed without stopping the aeration.

**Figure 7:**
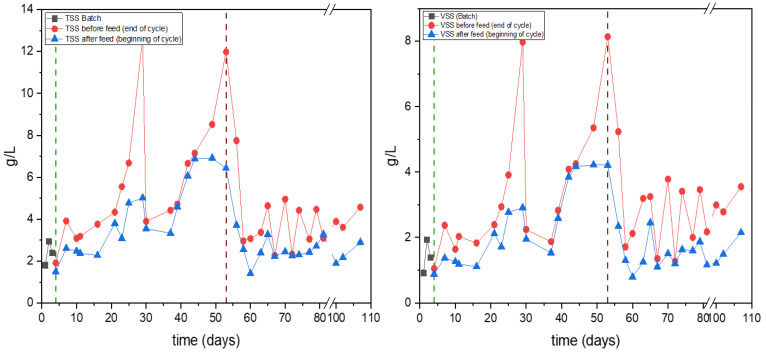
TSS (**left**) and VSS (**right**) concentrations during the SBR operation over time in the experiments assessing the impact of the settling phase. The green dashed vertical line indicates the operational shift from batch mode to the operational mode with settling phase, while the red one reflects the one in which the settling phase was removed from the cycles.

The concentration of the biomass over time during the operation of the SBR is presented in Figure 7. Measurements taken at the beginning and end of each cycle are distinguished in order to emphasize the variation in this operational scheme. More particularly, during the first operational phase that included settling, a trend of increasing suspended solids is observed. Both TSS and VSS concentrations increased gradually, reaching peak values of 12.6 g/L and 8.1 g/L, respectively. After removal of the settling phase (Period 2), i.e., on day 53, a sharp decrease in solids concentrations was observed. The absence of settling led to increased biomass washout, reflected by lower solids concentrations at the beginning of the cycle and sharper variations between feed and end-of-cycle measurements. In particular, TSS and VSS values exhibited strong fluctuations, with average concentrations of 3.3 ± 1.3 g/L and 2.1 ± 1.1, stabilizing at lower levels compared to Period 1, where settling was included. Similar washout effects in SBR systems have been reported when settling phases were shortened or omitted, leading to reduced solids retention. However, the VSS/TSS ratio was on average 0.57 ± 0.09 during period 1, and 0.63 ± 0.05 in period 2, suggesting that the microbial biomass that developed during the second period was metabolically more active.

**Figure 8:**
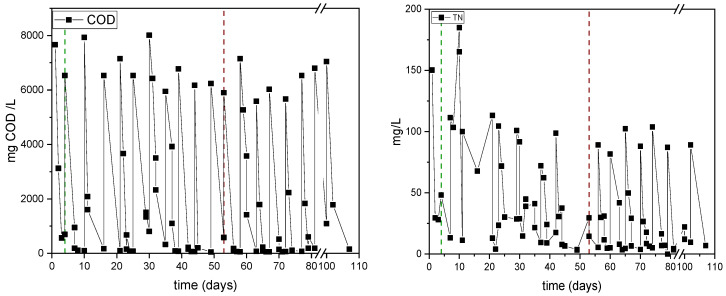
Monitoring of sCOD and TN concentrations during the SBR operation in the experiments assessing the impact of the settling phase. The green dashed line indicates the transition from batch to typical SBR mode (Period 1) and the red from typical SBR to operation without a settling phase (Period 2).

The pattern of sCOD and TN concentrations in the SBR for the whole operational time is shown in Figure 8. The average value of the influent sCOD (6.5 ± 0.6 g/L) remained the same across both operational modes. In Period 1, the effluent sCOD remained low, with an average removal efficiency of 87%. This cyclic removal reflects the well-established design of SBR processes, in which the settling phase ensures separation of biomass and stabilizes effluent quality. During period 2 (days 53–110), the settling step was removed, yet sCOD removal remained consistently high (93%), indicating that the microbial consortia were resilient and capable of maintaining organic matter degradation under continuous aeration and direct withdrawal conditions.

TN concentrations over time are presented on the right of Figure 7. In general, repeated drops during the nitrogen cycles in all the operational modes are shown. However, the nitrogen removal was not as effective as with the organic substrates. During Period 1, the average nitrogen consumption was 51%, and displayed great variability and intermittent spikes in the effluent TN. An intense peak emerging on day 10 was probably due to the release of nitrogen from cell lysis [45]. The microbial biomass may not have fully adapted to the new feed concentration and limitation conditions, thus limiting nitrogen uptake. In Period 2, the TN uptake (74%) presented a more stable pattern. Notably, the consistent nitrogen uptake observed in Period 2 under non-settling conditions maintained reliable nutrient limitation, which is a requirement for sustained PHA synthesis. This steady state suggests that continuous operation without settling not only preserves substrate removal efficiency but also creates favorable conditions for enhanced and steady polymer accumulation.

**Figure 9:**
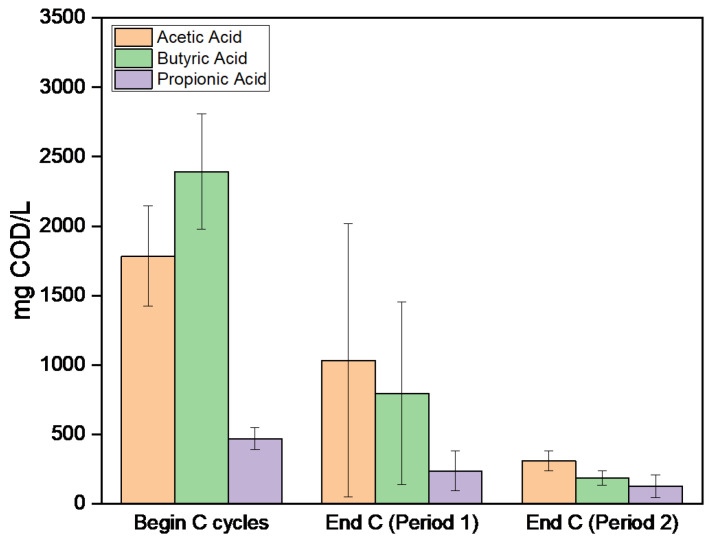
Average Concentrations of VFAs at the beginning and end of the c-cycle for both periods of SBR operation, in the experiments assessing the impact of the settling phase.

In Figure 9, the consumption of VFAs is presented. At the beginning of each carbon cycle, the average concentrations of acetic acid, propionic acid, and butyric acid were 1785 ± 362 mg COD/L, 469 ± 80 mg COD/L, and 2393 ± 414 mg COD/L, respectively. In Period 1, the consumption of acetic acid reached 42%. The average uptake of propionic and butyric acid at the end of the carbon cycles was 49% and 67%, respectively. This indicates the production of the PHBV polymer. Acetic acid, due to its property of being easily consumed by microorganisms as it is converted into acetyl-CoA and participates in the Krebs Cycle, producing energy for bacterial growth, is a particularly important component of the process. A greater reduction in the VFA concentrations is observed during Period 2. More specifically, the average consumption percentage of acetic acid rose to 83%, of propionic acid to 73% and finally, that of butyric acid was 92%. This explains that although the microbial strains are reduced due to their removal at the end of each cycle, the microorganisms that remain in the system are sufficient and capable of accumulating PHAs.

**Figure 10:**
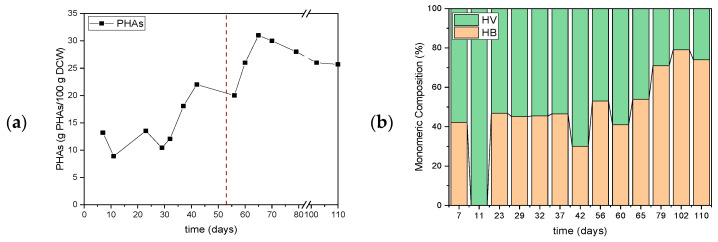
Yield of PHAs during each period of operation of SBR (**a**) and monomeric composition of the PHAs (**b**) in the experiments assessing the impact of settling phase.

The intracellular PHA accumulation is presented in Figure 10a. A progressive increase in polymer content over the course of the SBR operation, with a clear distinction between the two operational periods, is shown. During period 1, PHA concentrations fluctuated in the range 0.07–0.22 g PHA/100 g DCW, suggesting limited but consistent storage activity. This performance can be attributed to the conventional SBR scheme, where alternating feast and famine phases allow for moderate PHA accumulation, while biomass retention through settling maintains a balanced microbial consortium. In period 2, PHA concentrations increased sharply, reaching values up to 32 g PHA/100 g DCW. The absence of a settling phase likely promoted a selective pressure favoring PHA-accumulating bacteria capable of withstanding biomass washout, consistent with reports of enrichment strategies in feast-famine SBRs that enhance PHA yield [46]. This indicates that the modified operational scheme enhanced the metabolic shift towards PHA storage as a survival strategy under stress conditions. It is worth mentioning that most studies on PHA production without settling generally employ multi-reactor setups, as single-reactor SBRs without sedimentation are uncommon due to biomass washout [47].

The monomeric composition of the PHAs is presented in Figure 10b. With the exception of day 11, the percentages of HV and HB remained stable in Period 1, which indicates stability of the system during the biomass enrichment phase. HV dominated the copolymer structure, accounting for up to 60% of total monomers, consistent with the results of TSS and VSS, as well as COD and nitrogen consumption at the end of Period 1. In Period 2, where settling is omitted, a variation in the percentages is observed throughout this operational scheme, with an increasing trend in the HB content. Regarding the composition, the ratio achieved during this phase was HB:HV (62 ± 15):(38 ± 15). It is observed that the amount of HV decreases over time. This compositional shift may be linked to the operational changes induced by the removal of the settling phase, which likely altered substrate availability and microbial selection dynamics. Similar trends have been reported where feast–famine regimes and substrate composition strongly influence HB/HV ratios in mixed microbial cultures [36].

## 4. Conclusions

This study investigated PHA production in an SBR operated under different organic loadings and operational modes. The results reveal that operational conditions strongly influenced biomass stability, substrate utilization, and intracellular polymer accumulation.

Under the first experiment, moderate organic loading (Period 1) and complete nitrogen consumption supported stable biomass growth and higher intracellular PHA accumulation (11.74%), with a balanced HB:HV ratio that favored PHBV copolymer formation. At higher organic load (Period 2), nitrogen uptake was incomplete, biomass declined, and PHA yields decreased (5.27%), with production shifting towards HB-dominant polymers. In the second experiment, removal of the settling phase led to lower suspended solids concentrations due to biomass washout. However, this operational mode forced selective pressure that enriched for PHA-storing microorganisms. As a result, organic substrate removal remained efficient, nitrogen uptake improved, and intracellular PHA accumulation increased significantly when settling was omitted, reaching up to 32 g PHA/100 g DCW. Moreover, the copolymer composition shifted dynamically, with HV initially dominant but gradually giving way to HB-rich polymers as operation progressed.

These results highlight that both organic loading and mode of operation are critical for optimizing PHA production from food-waste-derived substrates. While the conventional SBR with settling ensures biomass retention and process stability at moderate organic loads, removing the settling phase can enhance selective enrichment of PHA-accumulating bacteria and increase polymer storage capacity under stress.

## Figures and Tables

**Table 1:** Operational Parameters of the SBR with settling phase and different organic loads.

Experimental Period	Days	C/N Ratio	sCOD (g/L)	TN (g/L)
Batch	5	100		
1	139	100	6.8 ± 1.4	0.06 ± 0.02
2	33	100	12.1 ± 2.9	0.12 ± 0.04

**Table 2:** Operational Parameters of the SBR to test the effect of including or not including a settling phase.

Experimental Period	Days	sCOD (g/L)	TN (g/L)	Operation Mode
Batch	4			
1	49	6.5 ± 0.7	0.08 ± 0.01	Settling phase
2	57			No Settling Phase

**Table 3:** Mean uptake of main carbon sources (expressed as COD equivalents) of the synthetic feed during the two experimental periods of the SBR in the experiment assessing the effect of organic load.

Experimental Period	Carbon Source	Mean COD Reduction (%)
1	Acetate	88.4 ± 29.8
	Butyrate	80.5 ± 29.2
	Propionate	85.2 ± 30.8
	EtOH	89.0 ± 22.5
	Glucose	100 ± 20.4
	Lactic Acid	96.0 ± 20.4
2	Acetate	77.8 ± 33.3
	Butyrate	78.5 ± 43.6
	Propionate	83.6 ± 32.6
	EtOH	100 ± 3.7
	Glucose	100 ± 23.8
	Lactic Acid	100 ± 24.2

## Data Availability

The original contributions presented in this study are included in the article. Further inquiries can be directed to the corresponding author.

## Abbreviations

The following abbreviations are used in this manuscript:

PHA	Polyhydroxyalkanoates
SBR	Sequencing Batch Reactor
3HB	3-R-hydroxybutyrate
3HV	3-R-hydroxyvalerate
PHBV	Poly (3-R-hydroxybutyrate-co-3-R-hydroxyvalerate)
MT	Metric ton
MMC	Mixed microbial cultures
FAO	Food and Agricultural Organization
DO	Dissolved Oxygen
sCOD	Soluble Chemical Oxygen Demand
TSS	Total Suspended Solids
VSS	Volatile Suspended Solids
VFAs	Volatile Fatty Acids
HPLC	High Performance Liquid Chromatography
TN	Total Nitrogen
DFR	Draw and Fill Reactor
PHB	Polyhydroxybutyrate
PHV	Polyhydroxyvalerate
